# Pulmonary Embolism Detection in Unenhanced CT Exams: A Radiomic-Based Classifier as a Promising Screening-Diagnostic Tool

**DOI:** 10.3390/jpm15100498

**Published:** 2025-10-17

**Authors:** Diletta Cozzi, Alessio Gnerucci, Giulia Zantonelli, Matteo Benelli, Maurizio Bartolucci

**Affiliations:** 1 Department of Emergency Radiology, Careggi University Hospital, 50127 Florence, Italy; 2 Department of Physics and Astronomy, University of Florence, 50127 Florence, Italy; 3 Department of Biomedical Sciences for Health, University of Milan, 20019 Milan, Italy; 4 Department of Medical Oncology, Hospital of Prato, Azienda USL Toscana Centro, 59100 Prato, Italy; 5 Department of Experimental and Clinical Biomedical Sciences, University of Florence, 50127 Florence, Italy; 6 Department of Radiology, Hospital of Prato, Azienda USL Toscana Centro, 59100 Prato, Italy

**Keywords:** pulmonary embolism, computed tomography pulmonary angiography, texture analysis, radiomics, emergency

## Abstract

**Background**: Pulmonary embolism (PE) is a life-threatening condition in which early diagnosis and prompt treatment are essential to reduce morbidity and mortality. Recent advances in radiomics have introduced software tools for PE detection on CT pulmonary angiography (CTPA), but their application to non-contrast chest CT remains unexplored. **Methods**: We retrospectively identified 57 CTPA examinations performed between January 2020 and March 2023. Patients were randomly assigned to training and validation cohorts; an additional 14 PE-negative patients were included in the validation cohort. Spherical regions of interest (ROIs) were manually placed within emboli and within normal-flow pulmonary arteries. From each ROI, 836 radiomic features were extracted. Statistically significant features were identified using the Wilcoxon test. A logistic regression classifier was then developed to discriminate between embolus and control ROIs based on their radiomic signatures. **Results**: Of the 836 features, 242 (all derived from wavelet-filtered images) showed significant differences between embolus and normal-flow ROIs on unenhanced CT. The classifier demonstrated high diagnostic performance in the validation cohort, with an AUC of 0.87 (95% CI: 0.86–0.88) and an accuracy of 0.77 (95% CI: 0.76–0.79), with only a limited number of misclassified ROIs. **Conclusions**: This preliminary study demonstrates the potential of radiomics to identify thrombi on unenhanced chest CT scans, offering a promising avenue for the early detection of acute PE. The proposed model showed satisfactory diagnostic accuracy with low few misclassifications, supporting further validation in larger cohorts.

## 1. Introduction

Pulmonary embolism (PE) is a potentially life-threatening condition and represents the third leading cause of mortality among cardiovascular events [1]. The current annual incidence of PE second to venous thromboembolism (VTE) is steadily increasing, with reported rates ranging from 39 to 115 per 100,000 individuals. Despite this rising incidence, mortality has shown a decreasing trend in recent years, largely attributable to advances in early diagnosis and the prompt initiation of effective therapies [2,3]. Indeed, early anticoagulation treatment has been shown to dramatically reduce mortality rates, from approximately 30% to as low as 8% [4]. In this context, computed tomography pulmonary angiography (CTPA) has emerged as the diagnostic gold standard for PE. Over the past two decades, its use in emergency departments (EDs) has expanded more than 27-fold, reflecting its crucial role in acute patient management [5,6,7,8,9,10]. Nevertheless, while CTPA ensures high diagnostic sensitivity and specificity, it is not without drawbacks. Appropriate patient selection remains essential, as indiscriminate use of CTPA exposes individuals to unnecessary ionizing radiation and contrast media administration, which may entail additional risks. A careful and personalized integration of clinical and laboratory findings is therefore required to guide imaging decisions.

Another relevant clinical challenge is that many chest CT scans are frequently performed in the ED for other suspected conditions—such as pneumonia, pulmonary edema, or acute exacerbations of pulmonary fibrosis—which may mimic PE clinically and radiologically. As a result, a substantial number of embolic events may go undetected, particularly in unenhanced CT examinations where intravascular thrombi are not easily visualized. This highlights the need for novel diagnostic approaches capable of supporting radiologists in the early recognition of PE without requiring contrast administration.

In recent years, considerable research has been devoted to the application of artificial intelligence techniques, especially deep learning and convolutional neural networks, to CTPA images. These methods have demonstrated promising results in the automated detection of endovascular thrombi, with the potential to improve diagnostic performance, streamline workflow, and reduce radiologists’ workload [11,12,13,14]. A well-trained neural network can further assist healthcare providers by prioritizing exams with suspected PE in the reporting worklist, thereby expediting diagnosis and communication, ultimately leading to improved clinical outcomes [15,16,17]. However, despite these advances, no studies to date have investigated the use of radiomics analysis for thrombus identification in unenhanced CT examinations. Developing such a model would be of particular value in emergency settings, where time is a critical factor and non-contrast CT is often the first imaging modality performed. In this preliminary study, we sought to develop and validate a radiomic model for the early detection of pulmonary emboli on non-contrast chest CT scans. Our aim was to provide radiologists with a high-performance, reproducible, and clinically applicable tool to support the triage of patients with suspected acute PE, potentially extending the diagnostic utility of routine unenhanced CT imaging.

## 2. Materials and Methods

### 2.1. Patients’ Selection

This is a retrospective and monocentric study, approved by the Ethics Committee of our Institution (AOU Careggi–ref. CEAVC 15671_oss; date of approval: 8 October 2019). Informed consent was obtained from all subjects involved in the study. The study group included patients with PE diagnosed via CTPA in our Emergency Radiology Department within the period January 2020–March 2023. The inclusion criteria were as follows: (1) acute PE with CT-confirmed main pulmonary vessel occlusion; (2) CTPA performed on the same CT scanner; (3) no thrombolysis before and/or during the CT acquisition. The exclusion criteria were (1) severe image artifacts (e.g., due to implants or motion artifacts); (2) no available unenhanced CT; (3) patients with segmental or subsegmental pulmonary embolism; (4) thrombolysis before CT imaging; (5) chronic PE; (6) patients younger than 18 years old. Finally, 57 patients met the inclusion criteria and constituted the study cohort. Extraction of datasets was performed utilizing the hospital Picture and Archiving Communication System (PACS). Clinical data including sex, age and risk factors were recorded.

### 2.2. CT Acquisition and Imaging Analysis

CT exams were performed with the same 128-row multidetector CT scan (Somatom Definition AS 128, Siemens, Erlangen, Germany) using helical acquisition and a pitch 0.9. The gantry rotation time was 0.5 s, and the beam collimation was 128 × 0.6 mm. The tube voltage was automatically chosen based on the acquired topogram (CAREkV, Siemens Healthcare), with the reference tube voltage set at 120 kVp. Real-time anatomy-based automated exposure control modulated the tube current (CARE-DOSE4D, Siemens Healthcare), with the reference tube current-time product set at 150 mAs. The scans were obtained in a supine position, with arms up, and in a caudo-cranial direction. The study protocol consisted of a baseline acquisition followed by the arterial phase. The iodinated contrast medium (35–40 mL of Iomeprol 400, 400 mg iodine/mL) was administered at a flow rate of 4 mL/s and a bolus tracking technique (Care Bolus; Siemens) was used for optimal opacification of the pulmonary arteries. The acquisition parameters for the basal scan were matrix size 512 × 512 pixels with slice thickness 2 mm, 120 kVp, 106 ± 44 mAs; for contrast-enhanced scans, the acquisition parameters were matrix size 512 × 512 pixels with slice thickness 1 mm, 120 kVp, 108 ± 42 mAs. Transverse images were contiguously reconstructed at 1 mm using standard filtered back-projection, a smooth reconstruction kernel (B30f) for mediastinal structures, and a sharp one (B70f) for parenchymal evaluation.

**Figure 1:**
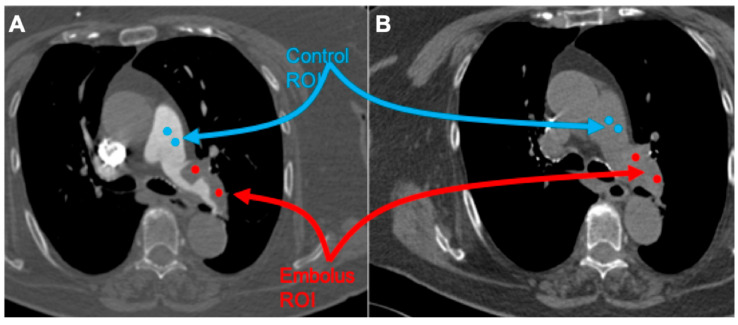
Representative case of the patient sample with acute PE with thrombus of the main left pulmonary artery. (**A**): Embolus (red)/control (blue) spherical ROI placed on contrast-medium-enhanced image. (**B**): Embolus (red)/control (blue) spherical ROI placed on the unenhanced image using the enhanced image as a reference.

All studies were reviewed by three radiologists, with 4, 8, and more than 20 years of experience in thoracic imaging. Embolus localization was classified as pulmonary trunk, main pulmonary arteries, lobar, and segmental arteries, according to the most proximally located embolus. The emboli regions were delineated by two radiologists (with 4 and 8 years of experience in thoracic imaging, respectively) and subsequently verified by a senior radiologist (with over 20 years of experience in thoracic imaging). CT images were processed with the free available texture analysis software 3DSlicer version 4.10.2 (open-source software; https://www.slicer.org/, accessed on 1 January 2024) [18]. Couples of 5 mm diameter spherical region of interest (ROI) were placed within the largest representative slide of the pulmonary embolus and normal flow vessel as follows (Figure 1):

(1) Embolus/Control ROI placed on contrast-medium-enhanced image;
(2) Embolus/Control ROI placed on the unenhanced image using the enhanced image as a reference.

The ROI was drawn clearly within the margin of the embolus with at least a 2 mm distance to the surrounding contrast media to address possible artifacts. A total of 836 features were extracted using the ‘radiomics’ extension of 3Dslicer [19]. All features were calculated for each ROI of each patient and a radiomic database was created, containing, in each column, the values of all features with the ROI and patient label. In this study, shape features were not considered because all ROIs have the same form. To test whether radiomic features had different values for embolus ROIs compared to control ROIs, the two groups of embolus and control ROIs extracted from the training cohort were compared using the Wilcoxon signed rank test. Features characterized by a Wilcoxon *p*-value < 0.05 can thus be indicated as those potentially differentiating embolus ROIs from control ROIs.

### 2.3. Model Development and Validation

Radiomic features represent a massive and redundant set of parameters, as many features can be, by their mathematical definition, statistically dependent. Therefore, the training of a classifier based on radiomic features must take this fact into account. To avoid overfitting on a set of redundant parameters, a classifier model based on logistic regression with ℓ2 regularization and regularization strength 10 (implemented with python package Scikit Learn) applied to the z-score-normalized radiomic features of radiomic features was developed to predict the clinical outcome of the ROI as an embolus or control [20]. The regularization strength parameter was therefore set to 10^4^ to select approximately 20 features out of the total 836. This regularization strength value was obtained by minimizing the model error across the defined grid, thereby ensuring stability and reproducibility of the results. Initially, the classifier was trained on the training cohort, and subsequently, the best-fit model was used to predict clinical outcomes in the validation cohort. Alongside the radiomic database, the model takes as input a clinical database containing, in each column, the clinical outcome of the ROI (1 for embolus and 0 for control). The clinical database also contains the ROI label, the patient label, and some technical parameters about the CT image (e.g., kV, exposure, acquisition kernel, and CTDI) that has been used to deeper investigate the dependence of model performances on such parameters. The classifier’s performance was evaluated by calculating several metrics typically used in classification tasks: the ROC curve and area under the ROC curve (AUC), the confusion matrix, precision, recall, f1-score, accuracy, specificity, sensitivity, and Cohen-k. For ROC curves and for each of the evaluated scores, confidence intervals were calculated, splitting the training set in 10 folds, training the model in each fold, evaluating all the model scores both for that training set fold and for the validation set, and finally calculating the mean and standard deviation of the 10 values of each score.

## 3. Results

A total of 57 patients with PE (male-to-female ratio 0.96; females 51%; median age 69.5; range 28–98 years) were included in our analysis. This patient set was randomly divided into training (*n* = 38, 66.7%) and validation (*n* = 19, 33.3%) cohorts. An additional 14 patients who tested negative for PE were then added to the validation cohort of 19 patients, resulting in a total of 33 patients in the validation cohort. A total of 238 ROIs (119 emboli + 119 controls) were considered in the cohort of 57 patients who tested positive for PE; of these, 162 ROIs (81 emboli + 81 controls) were included in the training set sample (*n* = 38). The validation set included a total of 120 ROIs, with 38 emboli and 38 controls from the 19 positive patients in the validation cohort, and 44 control ROIs from the cohort of 14 patients who tested negative for PE. Additionally, the 44 control ROIs from the 14 negative patients’ dataset were investigated separately.

### 3.1. Radiomic Features and Characterization

**Figure 2:**
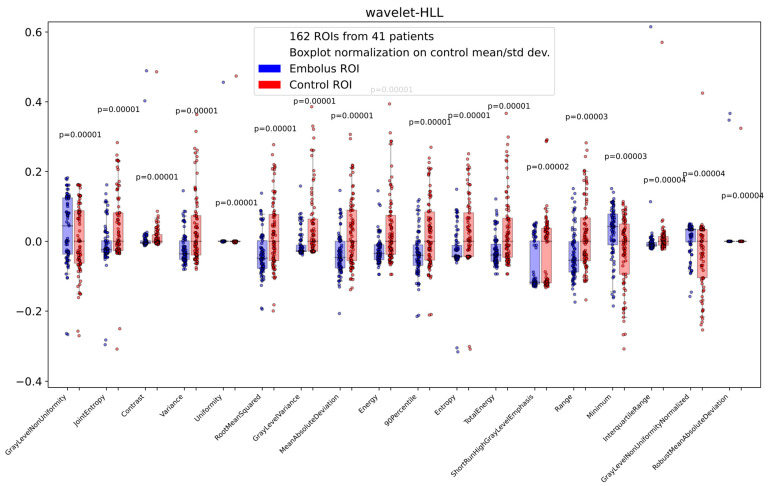
Exemplary box plots of the distributions on the embolus ROIs and control ROIs of all the wavelet-HLL features with *p*-value < 0.05. This feature class was the one with the lowest Wilcoxon *p*-values. Boxes represent the 25–75 percentile range of distributions while whiskers are 5–95 percentiles. Blue and red box plots represent embolus and control ROIs, respectively. Blue and red dots represent each feature value on the 81 embolus ROIs and 81 control ROIs, respectively.

Wilcoxon’s test on the distribution of each radiomic feature for the 81 embolus ROIs and 81 control ROIs extracted from the training cohort shows that 242 radiomic features from 836 have a Wilcoxon *p*-value < 0.05 (Figure 2 and Table 1). All the significant features were from wavelet-filtered images, with 66 belonging to the Gray-Level Co-occurrence Matrix (GLCM) class, 55 to the first-order class, 46 to the Gray-Level Run Length Matrix (GLRLM) class, 34 to the Gray-Level Size Zone Matrix (GLSZM) class, 29 to the Gray-Level Dependence Matrix (GLDM) class, and 12 to the Neighbouring Gray Tone Difference Matrix (NGTDM) class. This result indicates that these features have significantly different distributions in embolus ROIs compared to control ROIs and may therefore differentiate emboli from normal-flow vessels in unenhanced CT images.

### 3.2. Radiomic Classifier Model

**Figure 3:**
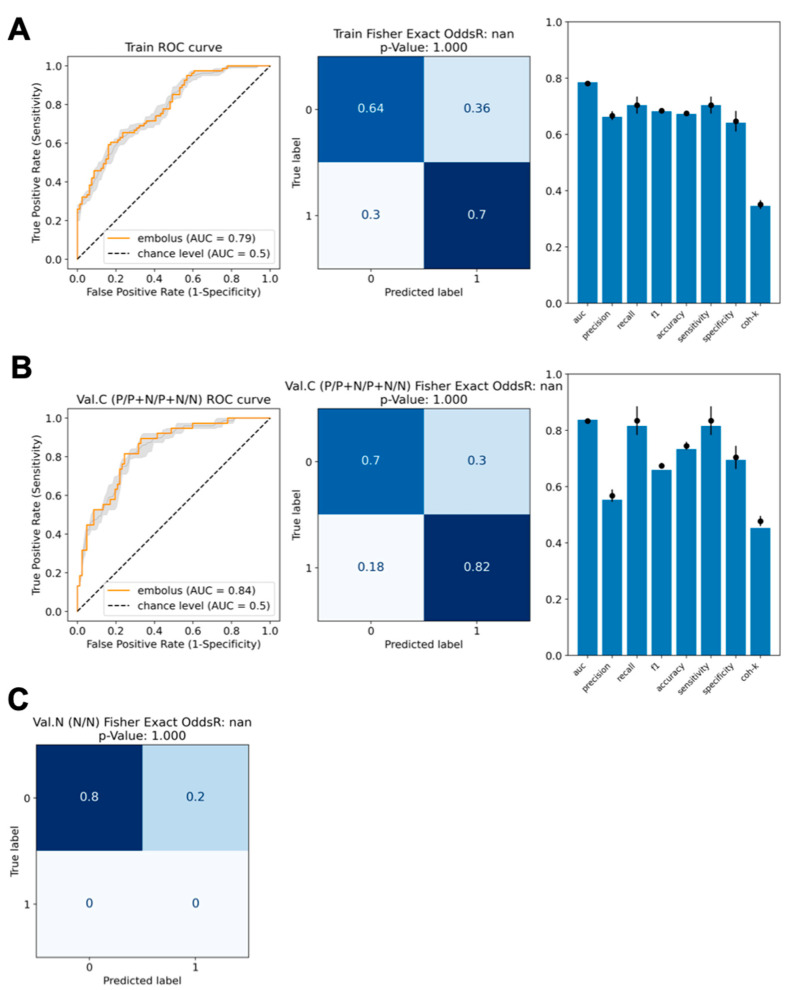
(**A**). Training set classifier model performance. (**B**). Validation set classifier model performance. (**C**). Contingency table of the classifier model on the validation set of 44 negative ROIs. On left panels, the ROC curve (orange line) with its confidence interval (gray thin line and shaded area) is shown, on the central panel the contingency table, and on the right panel the model scores (blue bars) together with the relative confidence intervals (black dots with error bars). All confidence intervals are evaluated by splitting the training set in 10 folds, training the model in each fold, evaluating all the model scores both for the training and validation sets, and finally calculating the mean and standard deviation of each score.

The performance of the radiomic classifier model is reported in Figure 3. In the training set, the model showed the following performance: AUC and accuracy of 0.91 (95% CI: 0.90–0.92) and 0.84 (95% CI: 0.83–0.85), respectively. False negatives (FNs) account for 20% of 81 positive ROIs, and false positives (FPs) account for 9.9% of 81 negative ROIs. In the validation set, AUC and accuracy showed values of 0.87 (95% CI: 0.84–0.88) and 0.77 (95% CI: 0.76–0.79), respectively. FNs account for 29% of 38 embolus ROIs, and FPs account for 13% of 82 control ROIs.

**Figure 4:**
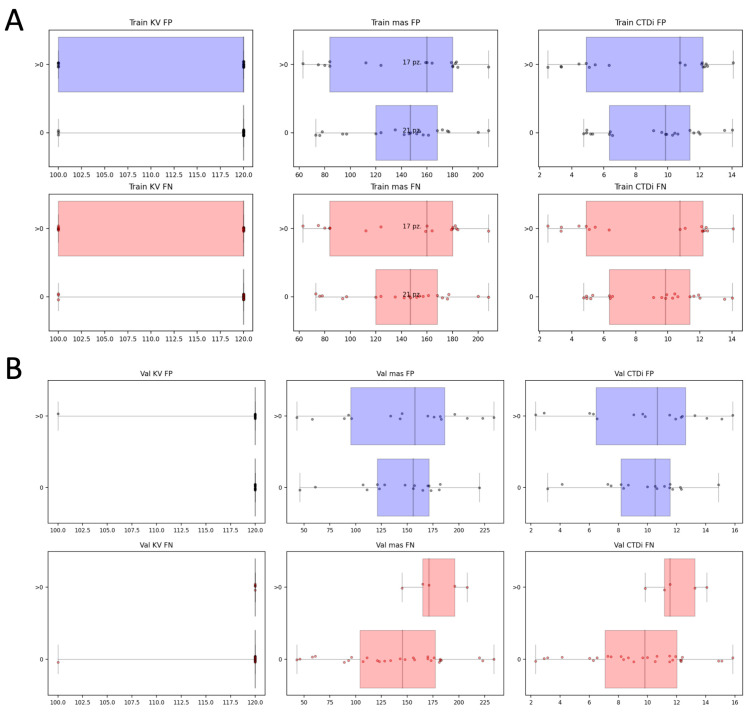
Distribution and box plots of KV, mas, and CTDI of the images (from left to right) as a function of the number of misclassifications produced by the model in each image (0 and >0 misclassifications). Blue dots and box plots represent FPs, and red dots and box plots represent FNs. On panels (**A**,**B**) are shown results for the training set and validation set, respectively.

We also conducted a comparative study on of the performance of different models based on logistic regression, support vector machine, and random forest classifier with or without z-score radiomic features normalization (Table S1). The scores of the three best performing models on the validation set are reported together with their confidence intervals. The scores of the different models are almost consistent, indicating that the predictive capability of radiomics for embolus identification is only marginally dependent on the type of the machine learning model. Finally, we investigated whether model misclassifications were related to the technical characteristics of the CT image (KV, mas, and CTDI) (Figure 4). We observed that the image noise pattern may influence the extraction and calculation of radiomic features; however, in this study, the distribution of KV, mAs, and CTDI of the images with misclassified ROIs (FN or FP) was consistent with those of the images with no misclassified ROIs (all the Wilcoxon *p*-values were >0.05; in particular, they ranged from 0.14 to 0.55 and from 0.14 to 0.73 in the training set and validation set, respectively), thus indicating that the performance of the model is not affected by these image technical characteristics.

## 4. Discussion

This preliminary study suggests that radiomics analysis for thrombus identification in unenhanced CT exams is useful for the early detection of acute PE. Moreover, the radiomic model developed here has satisfactory diagnostic accuracy values, with a low rate of misdiagnosed exams. In fact, this tested high-performance tool may support targeted triage in patients presenting with clinical features suggestive of acute PE. Acute pulmonary embolism is a prevalent condition that is often misrecognized and therefore potentially life-threatening in emergency patients [21,22]. Moreover, the increased use of imaging has made it extremely difficult for the healthcare system and radiologists to make a timely and reliable diagnosis of PE. Therefore, an accurate, precise, and timely diagnosis of PE is crucial to improve prognosis [23,24].

Regarding thrombus imaging, a plethora of investigations have been performed to better characterize thrombi in acute ischemic stroke patients and in portal vein thrombosis [25,26]. To date, clot-based radiomic studies have revealed and encouraged that clot radiomic features could help predict its composition [26]. Nevertheless, to date, there are no studies that apply radiomics analysis for thrombus identification in unenhanced CT exams. Our study confirms that radiomics analysis can provide information on the presence and microstructure of the embolus: the results we have obtained indicate that these features have significantly different distributions on the embolus ROIs compared to control ROIs and may therefore differentiate emboli from normal-flow vessels in unenhanced CT images.

Previous computer-aided detection (CAD) solutions for automatic PE detection are limited to CTPA studies [13,14,15,17,27]. The possibility of having a tool that allows clinicians and radiologists to rule out the suspicion of PE specifically in low-risk patients using unenhanced CT exams represents a diagnostic challenge. In this study, we identified that CT-based radiomic features could be strongly correlated with thrombus constituents and aimed to examine whether these features could predict the presence of PE on unenhanced CT scans alone. We developed a classifier model for PE detection and tested it on a clinical dataset containing 57 CT exams. The model achieved high accuracy and ROC in the detection of PE on unenhanced CT scans. Our model obtained an AUC of 0.84 and an accuracy of 0.75 in the validation set patient cohort with a limited number of misclassified ROIs (26% of FNs and 18% of FPs). Interestingly, the misclassifications produced by the model do not show any association with the KV, mAs, and CTDI of the CT image, indicating that classification performance is not influenced by such aspects of the CT scans. Therefore, the most common CT scan protocols already adopted for unenhanced exams are all equally suited for the aim of a radiomic-based PE classification. Based on our results, it would be interesting to apply our model as a screening test in patients undergoing unenhanced chest CT scans for reasons other than suspected PE, such as dyspnea, pneumonia, or chest pain, to alert on the possible concomitant presence of PE and potentially supplement the CT examination with contrast medium.

This study has several limitations: the first of these is the retrospective and monocentric nature of the work. Secondly, the patient sample is relatively small due to the single-center design. However, it was possible to develop a radiomics detection model even with the modest sample size included in the study. Since only emboli localized in the main pulmonary branches were selected, it is unclear whether this model is accurate in recognizing emboli in the more distal arterial branches; this aspect needs further investigation. In the future, it would be interesting to study the possibility to identify more distal and often misdiagnosed thrombi with radiomic analysis. In fact, future validation on segmental and subsegmental emboli, as well as across multiple institutions, will be necessary to confirm the robustness of our findings. Additional data must be collected to expand the subject cohort, enabling to strengthen the statistical evaluation and to improve the performance of the classifier. However, the use of this classifier model could be an important additional tool to be used in the emergency department, aiding in the detection of PE before contrast medium administration, especially in patients at risk of adverse events (such as those allergic to contrast medium or with reduced renal function) and in patients at low risk of PE.

In conclusion, our research underscores the considerable potential of radiomic analysis in facilitating the detection of PE in unenhanced CT exams. This non-invasive approach not only demonstrates robust diagnostic capabilities but also holds promise for reducing the necessity for extra scans, limiting the utilization of contrast agents, and helping radiologists and clinicians in the broad differential diagnosis of non-specific thoracic symptoms. The implications of our findings emphasize the significance of continued investigation and validation within clinical environments, which could pave the way for enhanced and more efficient techniques in the diagnosis and care of PE.

## Figures and Tables

**Table 1:** Scores of the three best performing of all the investigated models. Models scores on the validation set are reported together with their confidence intervals.

Model	AUC	Precision	Recall	F1	Accuracy	Sensitivity	Specificity	Cohen-k
Logistic regression l2 regularized; z-score normalization	0.869 [0.864, 0.874]	0.598 [0.574, 0.621]	0.884 [0.863, 0.905]	0.713 [0.693, 0.733]	0.774 [0.755, 0.794]	0.884 [0.863, 0.905]	0.723 [0.696, 0.750]	0.538 [0.503, 0.573]
Support vector machine; no normalization	0.896 [0.861, 0.932]	0.733 [0.671, 0.796]	0.585 [0.437, 0.734]	0.644 [0.545, 0.743]	0.800 [0.739, 0.861]	0.861 [0.753, 0.969]	0.90 [0.87, 0.93]	0.509 [0.373, 0.644]
Random forest classifier; no normalization	0.792 [0.709, 0.874]	0.580 [0.430, 0.730]	0.601 [0.514, 0.689]	0.581 [0.487, 0.676]	0.725 [0.652, 0.798]	0.601 [0.514, 0.689]	0.78 [0.68, 0.89],	0.378 [0.235, 0.521]

## Data Availability

The datasets used and/or analyzed during the current study are available from the corresponding author on reasonable request.

## Supplementary Materials

The following supporting information can be downloaded at: https://www.mdpi.com/article/10.3390/jpm15100498/s1, Table S1: Summary of the 242 radiomic features which were significantly different between embolus and control ROIs according to the Wilcoxon test. Features are presented in ascending order of Wilcoxon *p*-value adjusted with Benjamini–Hochberg *p*-value correction for multiple hypothesis testing. The right group of 4 columns is the continuation of the left group, always in ascending *p*-value order.

## Abbreviations

CTPA	computed tomography pulmonary angiography
ED	emergency department
GLCM	Gray-Level Co-occurrence Matrix
GLDM	Gray-Level Dependence Matrix
GLRLM	Gray-Level Run Length Matrix
GLSZM	Gray-Level Size Zone Matrix
NGTDM	Neighbouring Gray Tone Difference Matrix
PE	pulmonary embolism
ROI	region of interest
VTE	venous thromboembolism
