# Supplementary material for: Pulmonary Embolism Detection in Unenhanced CT Exams: A Radiomic-Based Classifier as a Promising Screening-Diagnostic Tool
Source: J Pers Med. 2025 Oct 17;15(10):498. doi: 10.3390/jpm15100498 (PMC12565646; doi:10.3390/jpm15100498)
Supplement: Supplementary file 1 [file jpm-15-00498-s001.zip › jpm-3861726-supplementary.pdf]

Image type	Feat. Class	Feat. Name	Adjusted p-val.	Image type	Feat. Class	Feat. Name	Adjusted p-val
wavelet-HLL	gldm	GrayLevelNonUniformity	0,000009	wavelet-HLL	glcm	Id	0,000197
wavelet-HLL	glcm	JointEntropy	0,000009	wavelet-LLH	gldm	SmallDependenceHighGrayLevelEmphasis	0,000202
wavelet-HLL	glcm	Contrast	0,000009	wavelet-LLH	gldm	LargeDependenceHighGrayLevelEmphasis	0,000212
wavelet-HLL	firstorder	Variance	0,000009	wavelet-LLH	glcm	DifferenceVariance	0,000219
wavelet-HLL	firstorder	Uniformity	0,000009	wavelet-HLL	gldm	HighGrayLevelEmphasis	0,000219
wavelet-HLL	firstorder	RootMeanSquared	0,000009	wavelet-HLL	glcm	ClusterProminence	0,000230
wavelet-HLL	gldm	GrayLevelVariance	0,000009	wavelet-HLL	firstorder	Maximum	0,000246
wavelet-HLL	firstorder	MeanAbsoluteDeviation	0,000009	wavelet-LLH	gldm	HighGrayLevelEmphasis	0,000248
wavelet-HLL	firstorder	Energy	0,000009	wavelet-LLH	glrlm	ShortRunHighGrayLevelEmphasis	0,000255
wavelet-HLL	firstorder	90Percentile	0,000009	wavelet-HLL	glrlm	RunLengthNonUniformityNormalized	0,000267
wavelet-LLH	firstorder	Energy	0,000009	wavelet-LLH	glcm	Contrast	0,000293
wavelet-LLH	glcm	Imc1	0,000009	wavelet-LLH	glcm	JointAverage	0,000314
wavelet-LLH	firstorder	RootMeanSquared	0,000009	wavelet-LLH	glszm	GrayLevelNonUniformityNormalized	0,000314
wavelet-LLH	firstorder	TotalEnergy	0,000009	wavelet-LLH	glszm	SmallAreaHighGrayLevelEmphasis	0,000314
wavelet-HLL	firstorder	Entropy	0,000009	wavelet-LLH	firstorder	Range	0,000314
wavelet-HLL	firstorder	TotalEnergy	0,000011	wavelet-LLL	glrlm	GrayLevelNonUniformity	0,000323
wavelet-HLL	glrlm	ShortRunHighGrayLevelEmphasis	0,000023	wavelet-HLL	glrlm	RunLengthNonUniformity	0,000354
wavelet-HLL	firstorder	Range	0,000026	wavelet-LLH	glcm	DifferenceEntropy	0,000371
wavelet-HLL	firstorder	Minimum	0,000026	wavelet-LLH	glrlm	HighGrayLevelRunEmphasis	0,000382
wavelet-LLH	glcm	Imc2	0,000027	wavelet-LLH	glcm	SumAverage	0,000466
wavelet-LLH	glcm	InverseVariance	0,000035	wavelet-LLL	gldm	GrayLevelVariance	0,000466
wavelet-HLL	firstorder	InterquartileRange	0,000035	wavelet-HLL	glrlm	GrayLevelNonUniformity	0,000499
wavelet-LLH	ngtdm	Complexity	0,000035	wavelet-LLH	ngtdm	Busyness	0,000524
wavelet-HLL	glrlm	GrayLevelNonUniformityNormalized	0,000035	wavelet-LLL	firstorder	Variance	0,000539
wavelet-HLL	firstorder	RobustMeanAbsoluteDeviation	0,000037	wavelet-LLH	glcm	Idm	0,000552
wavelet-HLL	glrlm	GrayLevelVariance	0,000040	wavelet-LLH	glcm	DifferenceAverage	0,000552
wavelet-HLL	gldm	SmallDependenceHighGrayLevelEmphasis	0,000048	wavelet-LLL	firstorder	MeanAbsoluteDeviation	0,000552
wavelet-HLL	glcm	DifferenceVariance	0,000049	wavelet-LLL	firstorder	Entropy	0,000578
wavelet-HLL	glcm	SumSquares	0,000050	wavelet-LLH	firstorder	Uniformity	0,000578
wavelet-LLH	gldm	GrayLevelVariance	0,000053	wavelet-LLL	glcm	Imc2	0,000578
wavelet-HLL	firstorder	10Percentile	0,000064	wavelet-HLL	glszm	HighGrayLevelZoneEmphasis	0,000635
wavelet-HLL	gldm	SmallDependenceEmphasis	0,000064	wavelet-LLL	glszm	LargeAreaEmphasis	0,000637
wavelet-HLL	glszm	GrayLevelVariance	0,000064	wavelet-HLL	glrlm	RunEntropy	0,000643
wavelet-LLH	glcm	ClusterProminence	0,000075	wavelet-LLH	glcm	SumEntropy	0,000688
wavelet-LLH	ngtdm	Strength	0,000081	wavelet-HLL	glszm	ZonePercentage	0,000741
wavelet-HLL	glcm	DifferenceEntropy	0,000081	wavelet-LLL	glrlm	GrayLevelNonUniformityNormalized	0,000741
wavelet-LLH	firstorder	Variance	0,000084	wavelet-HLL	gldm	LargeDependenceEmphasis	0,000741
wavelet-HLL	ngtdm	Complexity	0,000084	wavelet-HLL	glcm	ClusterTendency	0,000743
wavelet-HLL	glcm	DifferenceAverage	0,000084	wavelet-LLH	glcm	Autocorrelation	0,000794
wavelet-HLL	glrlm	HighGrayLevelRunEmphasis	0,000084	wavelet-HLL	glcm	MaximumProbability	0,000794
wavelet-LLH	glcm	MCC	0,000102	wavelet-LLH	firstorder	Mean	0,000794
wavelet-LLH	glszm	GrayLevelVariance	0,000103	wavelet-LLH	glrlm	ShortRunLowGrayLevelEmphasis	0,001011
wavelet-HLL	glcm	JointEnergy	0,000104	wavelet-LLH	glrlm	GrayLevelNonUniformity	0,001081
wavelet-LLH	glcm	ClusterTendency	0,000142	wavelet-LLH	glrlm	GrayLevelNonUniformityNormalized	0,001081
wavelet-HLL	glrlm	ShortRunEmphasis	0,000142	wavelet-LLL	glcm	DifferenceVariance	0,001081
wavelet-HLL	glcm	Idm	0,000142	wavelet-LLL	glcm	Imc1	0,001081
wavelet-LLH	glrlm	GrayLevelVariance	0,000142	wavelet-LLL	glcm	Contrast	0,001081
wavelet-HLL	glcm	SumEntropy	0,000142	wavelet-LLL	firstorder	Range	0,001113
wavelet-LLH	firstorder	10Percentile	0,000142	wavelet-LLH	firstorder	InterquartileRange	0,001201
wavelet-LLH	glcm	SumSquares	0,000143	wavelet-LLH	glszm	LowGrayLevelZoneEmphasis	0,001201
wavelet-LLH	glrlm	LongRunHighGrayLevelEmphasis	0,000143	wavelet-HLL	glszm	LargeAreaLowGrayLevelEmphasis	0,001236
wavelet-LLH	glrlm	RunEntropy	0,000150	wavelet-LLH	gldm	LowGrayLevelEmphasis	0,001397
wavelet-LLH	glszm	HighGrayLevelZoneEmphasis	0,000150	wavelet-LLH	glcm	JointEntropy	0,001465
wavelet-LLH	firstorder	MeanAbsoluteDeviation	0,000161	wavelet-LLL	firstorder	InterquartileRange	0,001465
wavelet-LLL	glrlm	RunEntropy	0,000161	wavelet-LLL	glcm	MCC	0,001466
wavelet-LLH	firstorder	Entropy	0,000162	wavelet-LLL	firstorder	RobustMeanAbsoluteDeviation	0,001509
wavelet-LLL	glrlm	GrayLevelVariance	0,000163	wavelet-HLL	glrlm	LongRunEmphasis	0,001524
wavelet-LLH	firstorder	RobustMeanAbsoluteDeviation	0,000174	wavelet-LLL	glcm	DifferenceEntropy	0,001539
wavelet-HLL	glrlm	RunPercentage	0,000197	wavelet-LLL	ngtdm	Strength	0,001555
wavelet-LLH	firstorder	Minimum	0,000197	wavelet-LLH	gldm	GrayLevelNonUniformity	0,001571

Table S1. Summary of the 242 radiomic features significantly different between embolus and control ROIs at the Wilcoxon test. Features are presented in ascending order of Wilcoxon p-value adjusted with Benjamini-Hochberg p-value correction for multiple hypothesis testing. The right group of 4 columns is the continuation of the left group always in ascending p-value order.

Image type	Feat. Class	Feat. Name	Adjusted p-val.	Image type	Feat. Class	Feat. Name	Adjusted p-val.
wavelet-HLH	firstorder	RobustMeanAbsoluteDeviation	0,001616	wavelet-LLL	glcm	InverseVariance	0,014187
wavelet-HLL	gldm	LargeDependenceLowGrayLevelEmphasis	0,001891	wavelet-LLH	glcm	MaximumProbability	0,014249
wavelet-LLL	glszm	ZoneVariance	0,001895	wavelet-LLL	glrlm	ShortRunHighGrayLevelEmphasis	0,014249
wavelet-LLL	glcm	DifferenceAverage	0,001895	wavelet-HHH	glrlm	LongRunHighGrayLevelEmphasis	0,014389
wavelet-LLL	glszm	LargeAreaLowGrayLevelEmphasis	0,001986	wavelet-LHH	glcm	MCC	0,014980
wavelet-LLL	glszm	SmallAreaHighGrayLevelEmphasis	0,002118	wavelet-HLH	glcm	DifferenceAverage	0,015358
wavelet-HLL	glszm	GrayLevelNonUniformityNormalized	0,002166	wavelet-HLH	firstorder	InterquartileRange	0,015746
wavelet-LLH	glrlm	LowGrayLevelRunEmphasis	0,002323	wavelet-HLL	glcm	Autocorrelation	0,016057
wavelet-LLH	glcm	Id	0,002329	wavelet-LHH	glcm	Imc2	0,016057
wavelet-LLL	glcm	SumSquares	0,002329	wavelet-LHH	glcm	Imc1	0,016215
wavelet-LLL	firstorder	Uniformity	0,002620	wavelet-HLL	glszm	ZoneVariance	0,016451
wavelet-HLH	firstorder	10Percentile	0,002791	wavelet-LLH	glrlm	RunPercentage	0,016451
wavelet-LLL	gldm	GrayLevelNonUniformity	0,002845	wavelet-HLL	glcm	JointAverage	0,016451
wavelet-LLL	glcm	ClusterTendency	0,003012	wavelet-LHH	firstorder	Minimum	0,016613
wavelet-LLL	glszm	GrayLevelVariance	0,003012	wavelet-LLL	firstorder	Maximum	0,018619
wavelet-HLH	firstorder	90Percentile	0,003012	wavelet-LLL	glrlm	HighGrayLevelRunEmphasis	0,019081
wavelet-LLL	glcm	Id	0,003043	wavelet-LLL	glrlm	LongRunHighGrayLevelEmphasis	0,019267
wavelet-LLH	gldm	SmallDependenceEmphasis	0,003332	wavelet-HHH	gldm	LargeDependenceHighGrayLevelEmphasis	0,019742
wavelet-LLL	gldm	SmallDependenceHighGrayLevelEmphasis	0,003332	wavelet-HLL	glcm	SumAverage	0,019935
wavelet-LLH	glszm	SizeZoneNonUniformity	0,003455	wavelet-HLL	glszm	SizeZoneNonUniformity	0,020512
wavelet-LLL	glcm	JointEntropy	0,003710	wavelet-HLH	glrlm	ShortRunEmphasis	0,020523
wavelet-LLH	glrlm	RunLengthNonUniformityNormalized	0,003813	wavelet-LLH	glrlm	ShortRunEmphasis	0,020523
wavelet-HLH	glcm	Contrast	0,003960	wavelet-HLH	glrlm	RunPercentage	0,020570
wavelet-HLH	glcm	Imc2	0,003960	wavelet-LLH	glszm	LargeAreaEmphasis	0,020622
wavelet-LLL	glcm	SumEntropy	0,004042	wavelet-LLL	gldm	HighGrayLevelEmphasis	0,021439
wavelet-HLL	ngtdm	Busyness	0,004042	wavelet-HLH	glrlm	GrayLevelVariance	0,022284
wavelet-HLL	ngtdm	Strength	0,004084	wavelet-LHH	firstorder	Range	0,023496
wavelet-LLL	glszm	ZoneEntropy	0,004162	wavelet-LLH	glszm	ZonePercentage	0,024308
wavelet-HLH	firstorder	MeanAbsoluteDeviation	0,004666	wavelet-LHH	firstorder	TotalEnergy	0,024647
wavelet-LLH	gldm	LargeDependenceLowGrayLevelEmphasis	0,004666	wavelet-HLL	glcm	Imc1	0,024884
wavelet-LLL	ngtdm	Complexity	0,004714	wavelet-LHH	firstorder	Energy	0,025123
wavelet-LLL	glrlm	RunLengthNonUniformity	0,005096	wavelet-LLH	glszm	LargeAreaHighGrayLevelEmphasis	0,027231
wavelet-LLL	ngtdm	Busyness	0,005414	wavelet-LHH	firstorder	Variance	0,027362
wavelet-HLL	glszm	LargeAreaEmphasis	0,005656	wavelet-HLL	glcm	MCC	0,027362
wavelet-LLH	glszm	SmallAreaEmphasis	0,006312	wavelet-HLL	glszm	LowGrayLevelZoneEmphasis	0,028349
wavelet-LLL	glszm	GrayLevelNonUniformityNormalized	0,006482	wavelet-HLL	gldm	LowGrayLevelEmphasis	0,028480
wavelet-HLL	glszm	SmallAreaHighGrayLevelEmphasis	0,006548	wavelet-HLH	glrlm	RunLengthNonUniformity	0,028822
wavelet-HLL	glszm	ZoneEntropy	0,006674	wavelet-LLH	glrlm	LongRunEmphasis	0,028822
wavelet-HLL	glcm	Imc2	0,007253	wavelet-LLL	glrlm	RunPercentage	0,029652
wavelet-LLL	glszm	HighGrayLevelZoneEmphasis	0,007326	wavelet-LLL	gldm	DependenceEntropy	0,029652
wavelet-LLH	glszm	ZoneEntropy	0,007339	wavelet-HLL	glrlm	LowGrayLevelRunEmphasis	0,029652
wavelet-HLH	firstorder	RootMeanSquared	0,007475	wavelet-LLL	glszm	ZonePercentage	0,030534
wavelet-LLH	glszm	LargeAreaLowGrayLevelEmphasis	0,007800	wavelet-LLL	gldm	SmallDependenceEmphasis	0,030642
wavelet-LLL	glcm	Idm	0,008008	wavelet-LLL	gldm	LargeDependenceEmphasis	0,031365
wavelet-LLH	glrlm	LongRunLowGrayLevelEmphasis	0,008767	wavelet-HLL	glrlm	LongRunLowGrayLevelEmphasis	0,032550
wavelet-HLH	firstorder	Variance	0,009439	wavelet-HLH	glrlm	RunLengthNonUniformityNormalized	0,032857
wavelet-LLL	glcm	ClusterProminence	0,009533	wavelet-LLH	gldm	LargeDependenceEmphasis	0,033166
wavelet-LLH	firstorder	Median	0,010098	wavelet-HLL	glrlm	LongRunHighGrayLevelEmphasis	0,033479
wavelet-LLH	glszm	ZoneVariance	0,010361	wavelet-LLL	gldm	LargeDependenceLowGrayLevelEmphasis	0,034261
wavelet-LLH	glcm	JointEnergy	0,010464	wavelet-LHH	firstorder	Entropy	0,034817
wavelet-LLL	glcm	JointEnergy	0,010568	wavelet-LHH	firstorder	RootMeanSquared	0,034908
wavelet-LLH	glcm	ClusterShade	0,010842	wavelet-HLH	gldm	LargeDependenceEmphasis	0,035568
wavelet-LLL	glrlm	ShortRunEmphasis	0,012145	wavelet-LLH	gldm	DependenceEntropy	0,035568
wavelet-HLH	firstorder	Energy	0,012145	wavelet-HLH	ngtdm	Complexity	0,036641
wavelet-HLH	glcm	Imc1	0,012264	wavelet-LHH	ngtdm	Contrast	0,040373
wavelet-LLL	ngtdm	Coarseness	0,012385	wavelet-LHL	glcm	Contrast	0,040745
wavelet-LLL	glrlm	RunLengthNonUniformityNormalized	0,012508	wavelet-HLH	gldm	GrayLevelVariance	0,041071
wavelet-HLH	firstorder	Maximum	0,012828	wavelet-HLH	firstorder	Range	0,044356
wavelet-HLH	glcm	MCC	0,012955	wavelet-LHL	glcm	Idm	0,047187
wavelet-LLH	glrlm	RunLengthNonUniformity	0,013701	wavelet-LHL	firstorder	MeanAbsoluteDeviation	0,049523
wavelet-HLH	firstorder	TotalEnergy	0,014049	wavelet-HLH	glszm	SizeZoneNonUniformity	0,049808

Table S1 (continuation). Summary of the 242 radiomic features significantly different between embolus and control ROIs at the Wilcoxon test. Features are presented in ascending order of Wilcoxon p-value adjusted with Benjamini-Hochberg p-value correction for multiple hypothesis testing. The right group of 4 columns is the continuation of the left group always in ascending p-value order.
